# The NIH BRAIN Initiative: Advancing neurotechnologies, integrating disciplines

**DOI:** 10.1371/journal.pbio.3000066

**Published:** 2018-11-26

**Authors:** Meghan C. Mott, Joshua A. Gordon, Walter J. Koroshetz

**Affiliations:** 1 National Institute of Neurological Disorders and Stroke, National Institutes of Health, Bethesda, Maryland, United States of America; 2 National Institute of Mental Health, National Institutes of Health, Bethesda, Maryland, United States of America

## Abstract

In 2014, the National Institutes of Health (NIH) began funding an ambitious research program, the Brain Research through Advancing Innovative Neurotechnologies (BRAIN) Initiative, with the singular focus of advancing our understanding of brain circuits though development and application of breakthrough neurotechnologies. As we approach the halfway mark of this 10-year effort aimed at revolutionizing our understanding of information processing in the human brain, it is timely to review the progress and the future trajectory of BRAIN Initiative research.

## Abstract 2

This Community Page article describes how the National Institutes of Health BRAIN Initiative offers an unprecedented opportunity to accelerate the development and application of new technologies to enable researchers to produce dynamic pictures of the brain, showing how individual cells and complex circuits interact in real time.

Throughout history, new tools have driven scientific revolutions in multiple disciplines [1]. Unraveling how the human brain processes information and supports a diverse spectrum of behaviors requires such a scientific revolution and an arsenal of next generation tools to be deployed by neuroscientists across the basic, translational, and clinical landscapes. Capturing the full record of neural activity or what has been called a “brain activity map” across spatial and temporal domains is a daunting technological challenge that needs to be met to achieve a more complete understanding of fundamental and pathological brain processes [2]. Launched on April 2, 2013, the key goal of the Brain Research through Advancing Innovative Neurotechnologies (BRAIN) Initiative is to develop innovative technologies to interrogate how the brain’s cells and circuits interact at the speed of thought and, ultimately, to reveal the complex links between brain function and behavior. Because brain architecture stretches from the scale of molecular interactions at the trillions of synapses to the billions of cell bodies that connect to form local networks that then integrate across multiple brain regions, the challenges are extraordinary. In addition to these spatial scales, there are temporal scales, as brain circuits are not static but continually change as a result of neural activity, developmental stage, and aging. Despite this complexity, the technologies emerging from the BRAIN Initiative are opening new doors to decipher how the brain records, processes, uses, stores, and retrieves vast quantities of information. They have the potential to facilitate a quantum leap toward understanding brain function and its disruption in disease, making circuit abnormalities the basis of diagnostics and the normalization of circuit function the target of future intervention in neuro/mental/substance-abuse disorders.

The BRAIN Initiative signals a paradigm shift for neuroscience, as it requires the scientific community to work together toward a neural network understanding of the brain. It is critically important to evaluate the BRAIN Initiative in the context of past and future advances in molecular and genetic neuroscience [3]. It is the ability to combine molecular, genetic and now systems level measures that paves the way for exciting possibilities in building a more complete narrative of how the brain functions in health and disease. Many participants from public and private sectors have joined in pursuit of BRAIN Initiative goals. These include federal agencies: National Science Foundation (NSF), Food and Drug Administration (FDA), Defense Advanced Research Projects Agency (DARPA), and Intelligence Advanced Research Projects Agency (IARPA), as well as private industry, philanthropists, nonprofit organizations, foundations, colleges, and universities. A BRAIN Initiative Alliance was formed to coordinate among a host of efforts. In addition, BRAIN has blossomed into a global enterprise connecting scientists in countries around the world, including Canada, the European Union, China, Australia, Korea, and Japan. A newly established International Brain Initiative launched by the Kavli Foundation will promote coordination among these groups. Scientific advancement thrives when data and research are shared beyond borders, and it is clear that BRAIN succeeds best when it transcends conventional disciplinary boundaries and engages experts from fields beyond the traditional neurosciences, including physics, mathematics, statistics, engineering, chemistry, nanotechnology, and computer science [4]. Connecting the dots of discovery science from molecular interactions to cellular activity to the dynamic firing of large neuronal ensembles can only be achieved through a large-scale coordinated program held together by access to powerful instrumentation, high-quality data resources, and frequent dialogue among scientists from varying disciplines. To these ends, BRAIN funds training and technology dissemination, data standards, archives, and analytics, as well as convenes its scientists in workgroups and at an annual investigator meeting.

To help guide the BRAIN Initiative effort, the National Institutes of Health (NIH) first established a high-level working group of the Advisory Committee to the Director (ACD) to develop a roadmap. Their report, *BRAIN 2025: A Scientific Vision*, provided a framework for a suite of bold funding opportunities outlined in a thoughtful multiyear scientific plan for the NIH BRAIN Initiative.

*BRAIN 2025* described seven areas of high priority for the NIH BRAIN Initiative, which are outlined here with a very brief note on progress [5].

1. Discovering diversity: identify and provide experimental access to the different brain cell types to determine their roles in health and disease. Characterization of all the cell types in the nervous system is within reach, and a cell “census” of neuronal and glial cell types will aid in the development of tools that can precisely target and manipulate them.
  The BRAIN Initiative Cell Census Network (BICCN), launched in 2017 based on extraordinary early progress, is the single largest BRAIN Initiative project to date and plans to invest US$250 million over the next five years to construct a comprehensive reference of diverse cell types in human, monkey, and mouse brains. This team science program will provide essential characterization for the diversity of cell types, an open-access 3D digital mouse brain cell reference atlas, and a comprehensive neural circuit diagram, as well as genomic access to specific cell types to monitor, map, or modulate their activity. These tools will serve as unprecedented resources for the scientific community to develop a deep understanding of neural circuit function across species. To achieve its goals, the BICCN requires its scientists to work together with agreed upon standards, methods, and a common framework for assembling the large variety of data coming from multiple laboratories, a social neuroscience experiment in itself.
2. Maps at multiple scales: generate circuit diagrams that vary in resolution, from synapses to the whole brain. As our ability to map neuronal connections within local circuits; micro-, meso-, and macroconnectomes; and between distributed brain systems improves, scalable circuit maps will relate structure to function.
  Successful efforts have been made in serial section electron microscopy and their analytic pipelines to enable whole brain connectomics in *Drosophila* and zebrafish as well as in regions of the mouse cortex. Improved track-tracing tools that leverage multiphoton imaging, expansion microscopy, and advanced techniques like viral circuit tracing have been devised and applied to identify synaptic inputs to specific neurons. Innovative whole brain–clearing techniques like clear lipid-exchanged acrylamide-hybridized rigid imaging/immunostaining/in situ hyrbridization-compatible tissue-hydrogel (CLARITY) enable morphological assessment in 3D and should be adaptable to postmortem human tissue.
3. The brain in action: produce a dynamic picture of the functioning brain by developing and applying improved methods for large‐scale monitoring of neural activity. Various neuronal-recording methods continue to emerge and offer innovative ways to record neuronal activity at a single-cell level from ever more cells over larger brain regions in awake behaving animals. Successful efforts have visualized network brain activity over wide areas of the mouse cortex while preserving the ability to focus at single-cell resolution. Investigator teams are recording longer and from more brain regions in patients undergoing deep brain stimulation to understand human circuit activity underlying speech, movement, intention, and emotion.
4. Demonstrating causality: link brain activity to behavior with precise interventional tools that change neural circuit dynamics. Tools that enable circuit manipulation open the door to probe how neural activity gives rise to behavior. Teams of scientists are monitoring and modulating neural activity linked to behavior both in awake behaving animals and in patients undergoing deep brain stimulation for disorders such as epilepsy, Parkinsons disease, chronic pain, obsessive compulsive disorders, and depression.
5. Identifying fundamental principles: produce conceptual foundations for understanding the biological basis of mental processes through development of new theoretical and data-analysis tools. The ability to capture neural activity in thousands to millions of neurons poses analytic challenges but offers a new opportunity to understand the language of the brain and the rules that underly its information processing. Progress relies on engaging statisticians, mathematicians, and computer scientists to join the effort. Teams of scientists are capturing neural activity in specific circuits and developing models that explain and predict key circuit characteristics as well as theories of fundamental brain processes.
6. Advancing human neuroscience: develop innovative technologies to understand the human brain and treat its disorders; create and support integrated human brain research networks. The burden of illness due to neurologic, psychiatric, and substance-abuse disorders is due largely to disordered brain circuits. The ability to measure and characterize activity in these circuits will change diagnostics and provide targets to precisely modulate them, ultimately changing the landscape of therapeutics. Early examples are the tuning of closed-loop deep brain stimulation devices based on measures of brain activity in patients with essential tremor and Parkinsons disease. Ultimately, the tools and technologies that emerge from BRAIN are expected to open the door to new FDA-approved drug therapies and devices for neurological and psychiatric disorders over the next decade.
7. From BRAIN Initiative to the brain: integrate new technological and conceptual approaches produced in Goals 1–6 to discover how dynamic patterns of neural activity are transformed into cognition, emotion, perception, and action in health and disease. Taken together, the goals outlined above lay the foundation for a comprehensive, integrated, and mechanistic understanding of brain function across species to understand the basis of our human capabilities. Since the NIH BRAIN Initiative began, more than 500 awards have gone out to investigators around the world, reflecting an investment of nearly US$1 billion.

A current listing of numerous active funding opportunities are posted regularly on the NIH BRAIN Initiative website, including those focused on large-scale recording and modulation of the nervous system; the biology and biophysics of neural stimulation; next-generation invasive devices; tools to target, identify, and characterize non-neuronal cells; and early stage next-generation human brain imaging. Upcoming funding opportunities are also highlighted, including one notice to support research on the fundamental neurobiology of pain processing. As the NIH rolls out a multipronged approach to address the nation’s opioid crisis, it is expected that the unique opportunities of the BRAIN Initiative will enable production of detailed maps of pain and reward circuits and the adoption of powerful new tools for monitoring and modulating pain and reward circuit activity, leading to significant advances in the understanding of pain and addiction.

Since its inception, the NIH has been actively working to integrate ethical considerations into BRAIN Initiative research [6]. *BRAIN 2025* outlined neuroethics goals, including providing resources for collecting and disseminating best practices for the conduct of ethical scientific research, particularly for the conduct of clinical research. The NIH funded its first set of neuroethics R01 research grants in fiscal year (FY)2017 to support efforts addressing core ethical issues associated with research focused on the human brain and resulting from emerging technologies and advancements supported by the BRAIN Initiative (the reissued funding opportunity can be found here: RFA-MH-18-500). The NIH published a recent notice encouraging investigators to apply for supplement funding to integrate neuroethics perspectives and approaches into existing BRAIN Initiative awards. Methods and research with ethical implications that merit thoughtful consideration are infused throughout BRAIN Initiative–funded research, with integrated activities between ethicists and neuroscientists.

Generating innovative tools to enable researchers to record signals from brain cells in greater numbers at faster speeds than ever before requires diverse, interdisciplinary teams of talented scientists approaching problems from multiple perspectives. Mathematical modeling, statistical analysis, and exploratory data mining are all powerful techniques that will be necessary to define the general principles of brain function that will have a profound impact on neuroscience. Attracting scientists from disparate fields into neuroscience, particularly from quantitative disciplines like engineering, physics, computational science, and chemistry, is crucial to the advancement of neurotechnology. Thus far, the BRAIN Initiative has funded hundreds of investigators across 12 scientific disciplines and, in FY16, funded as many engineers as neuroscientists. Still, collaborations of researchers from various disciplines will be essential in uncovering new discoveries and translating them to applications.

Looking ahead, the NIH is formally revisiting *BRAIN 2025*’s priorities to provide an updated scientific vision to guide the second half of the initiative through a new ACD BRAIN Initiative working group. The goals of this effort are to 1) review BRAIN Initiative activities and progress to date; 2) suggest changes to specific goals from the *BRAIN 2025* report in response to the evolving scientific landscape; 3) with the *BRAIN 2025* report as a guide, identify new opportunities for research and technology development, taking into consideration neuroethics guiding principles developed for human neuroscience research; and 4) consider unique opportunities for the BRAIN Initiative to train and empower the broader neuroscience research community. As with the initial *BRAIN 2025* report, community input will be an essential part of the process. Through May 2019, the working group will continue to accept questions and comments via email at BRAINfeedback@nih.gov, along with responses submitted through a recent request for information (RFI) as they draft their final report. A series of three targeted country-wide workshops were recently held to provide further opportunities for public commentary, and these can be viewed at https://www.braininitiative.nih.gov/about/acd-wg.htm.

The BRAIN Initiative offers a unique, unprecedented opportunity to accelerate the development and application of new technologies that will enable researchers to produce dynamic pictures of the brain, showing how individual cells and complex circuits interact in real time. As the beginning of a new technological era, this golden age of neuroscience promises to elucidate how the brain operates at multiple levels, from nano-scale molecules to meter-scale systems. Thanks to sustained congressional investment in the BRAIN Initiative appropriated through the 21st Century Cures Act, this transdisciplinary effort can continue to grow and be driven by an amalgamation of expertise from biochemistry to physics to mathematics for which researchers from all backgrounds contribute to understanding the human brain.

## Abbreviations

ACD: Advisory Committee to the Director
BICCN: BRAIN Initiative Cell Census Network
BRAIN: Brain Research through Advancing Innovative Neurotechnologies
CLARITY: clear lipid-exchanged acrylamide-hybridized rigid imaging/immunostaining/in situ hyrbridization-compatible tissue-hydrogel
DARPA: Defense Advanced Research Projects Agency
FDA: Food and Drug Administration
FY: fiscal year
IARPA: Intelligence Advanced Research Projects Agency
NIH: National Institutes of Health
NSF: National Science Foundation
RFI: request for information
